# *Ex vivo* imaging reveals neutrophil behaviors in the adult zebrafish heart

**DOI:** 10.1242/jcs.264418

**Published:** 2026-05-11

**Authors:** Pinelopi Goumenaki, Mridula Balakrishnan, Kenny Mattonet, Didier Y. R. Stainier, João Cardeira-da-Silva

**Affiliations:** ^1^ Department of Developmental Genetics, Max Planck Institute for Heart and Lung Research, 61231 Bad Nauheim, Germany; ^2^ DZHK German Centre for Cardiovascular Research, Partner Site Rhine-Main, 61231 Bad Nauheim, Germany; ^3^ Cardio-Pulmonary Institute (CPI), 61231 Bad Nauheim, Germany; ^4^ Imaging Platform, Max Planck Institute for Heart and Lung Research, 61231 Bad Nauheim, Germany; ^5^ University of Toulouse, CNRS UMR 5070, INSERM U1301, EFS, Institut RESTORE, 31100 Toulouse, France

**Keywords:** Dexamethasone, Lipopolysaccharide, Heart explant, Inflammation, Motility, Morphodynamics

## Abstract

Current approaches for investigating cells in mature internal organs of vertebrate model organisms focus primarily on cell morphology, numbers and spatial distribution using static images from fixed samples, failing to provide information on cell behavior. Here, we focus on the robust dynamics of neutrophils and analyze their motility and morphodynamic (i.e. time-resolved shape changes) behaviors in adult zebrafish hearts *ex vivo*. To modulate neutrophil activity, we exposed cryoinjured heart explants to dexamethasone (DEX) or lipopolysaccharide (LPS), anti- and pro-inflammatory drugs, respectively. Whereas DEX halted neutrophil motility and morphodynamics, LPS drastically increased them, emphasizing the suitability of this experimental setup for modulating and investigating cell behavior. Detailed analyses identified parameters predictive of cell activity and confirmed that temporal changes in morphological parameters, including two-dimensional measures, are stronger indicators of cell behavior than their corresponding mean values. Profiling neutrophil behavior during cardiac regeneration further revealed temporal variation of behavioral states and substantial heterogeneity at each time point. Altogether, we introduce the use of a cardiac explant system to analyze cell behaviors over time and present it as an additional platform for compound screening.

## Teaser

**Summary:** Dynamic *ex vivo* imaging of cryoinjured zebrafish hearts reveals that neutrophil motility and morphodynamics correlate with inflammatory activity, and uncovers behavioral state heterogeneity during regeneration.

## INTRODUCTION

Zebrafish is an excellent vertebrate model to investigate cell dynamics through live imaging approaches. The optical clarity of zebrafish embryos and larvae has enabled the study of motility and morphodynamic behaviors occurring during organogenesis (Shah et al., 2019), including in the nervous system (Kirby et al., 2006), vasculature (Gebala et al., 2016), liver (Cayuso et al., 2016), pancreas (Yang et al., 2018), ear (Munjal et al., 2021), and heart (Gunawan et al., 2019; Priya et al., 2020), among others. Advancements in imaging approaches (e.g. fast-acquisition high-resolution microscopy; Huisken and Stainier, 2009; Mickoleit et al., 2014; Saberigarakani et al., 2025) and genetic tools (e.g. cell labeling and tracking; Wen et al., 2021; Teranikar et al., 2024 preprint) have further boosted the 4D imaging and quantification of these cell behaviors during development, such as in the developing heart. However, at the onset of the juvenile stage, zebrafish lose their optical transparency, limiting live imaging applications to superficial structures, such as some blood vessels (Xu et al., 2014) and lymphatics (Castranova et al., 2021), or immune cells such as neutrophils (Castranova et al., 2022) and T-cells (Robertson et al., 2023) near the surface of the animals. Internal organs become largely inaccessible for imaging, even in transparent genetic backgrounds. Consequently, studies of internal adult organs mostly rely on snapshots of fixed wholemount and sectioned tissues. Although these studies provide valuable information on cell numbers, spatial distribution patterns and cell morphology, they fail to capture cellular dynamics, thereby limiting the analysis of motility and morphodynamic behaviors.

The adult zebrafish heart possesses a remarkable ability to regenerate, through processes involving the concerted action of multiple cell types (Poss et al., 2002; Ross Stewart et al., 2022). To overcome the technical limitations of imaging the adult zebrafish heart *in vivo*, a few studies have advanced the imaging of cardiac explants (Kikuchi et al., 2011; Cao and Poss, 2016). These studies have focused on cell types with relatively low kinetic activity, such as epicardial cells (Cao et al., 2017), as well as endocardial and endothelial cells (Kikuchi et al., 2011; Yip et al., 2020), providing insights into their migration and proliferative behaviors through timelapse imaging over extended periods of time. However, short-term motility and morphodynamic behaviors, which require fast-acquisition methods to detect rapid, fine and transient cell alterations in highly kinetic cells, such as immune cells, remain underexplored. Live imaging of cardiac slices has also been used to analyze cardiomyocyte behavior (Honkoop et al., 2021) and their interaction with macrophages (Constanty et al., 2025). Although valuable for studying particular biological processes, such as cell–cell interactions, this strategy has the limitation of introducing additional injuries (i.e. sectioning the live heart), to which immune cells might respond, thereby potentially inducing behavioral artifacts. In addition, its labor intensiveness limits the throughput for larger-scale analyses.

Immune cells, in particular neutrophils, are rapid responders to various cues, including those induced by tissue damage, such as heart injury (Puhl and Steffens, 2019; Zhang et al., 2024; Yang et al., 2025). In the regenerating zebrafish heart, neutrophils infiltrate the injured tissue as early as 6 h following cryoinjury (Lai et al., 2017). Although they play a role in processes such as macrophage polarization, revascularization and epicardial activation (Peterson et al., 2024), they are primarily responsible for the initiation of an early inflammatory wave required for the regenerative response (de Preux Charles et al., 2016). However, persistent inflammation associated with prolonged neutrophil retention within the injured tissue impairs cardiac regeneration (Lai et al., 2017; Xu et al., 2019), underscoring the importance of self-limiting inflammation and transient neutrophil infiltration. Although the roles of neutrophils have been extensively investigated during tissue homeostasis and regeneration, how their behaviors relate to their activities remains poorly understood.

Here, we analyze neutrophil motility and morphodynamic behaviors via live imaging of cryoinjured zebrafish heart explants. Importantly, through pharmacological immunomodulation assays, we show that these behaviors can be predictive of the inflammatory activity of neutrophils. We also identify behaviorally distinct neutrophil subsets across multiple stages of the cardiac regenerative process. Overall, our data reveal that dynamic measurements of motility and morphodynamic behaviors are strong predictors of immune cell activity, complementing static cell morphological and topological assessments. Moreover, this approach constitutes a valuable platform for immunomodulation trials, which are particularly relevant in the context of inflammation, cardiac injury and regeneration.

## RESULTS AND DISCUSSION

### Neutrophil behavior in cryoinjured cardiac ventricles is affected by immunomodulators

**Fig. 1.**
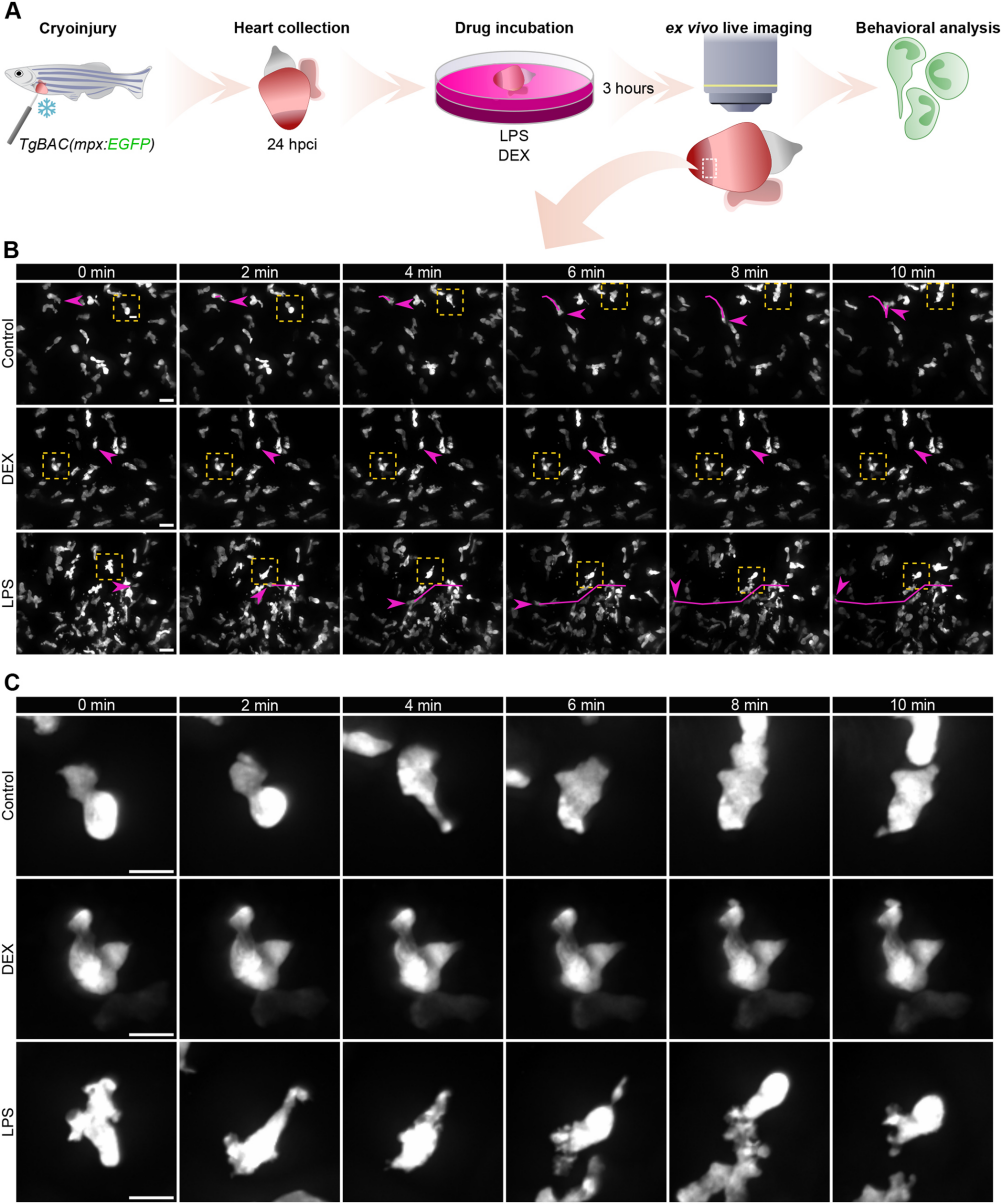
**Neutrophil behavior in cryoinjured cardiac ventricles is altered upon treatment with immunomodulators.** (A) Schematic illustration of the experimental setup used to image and analyze neutrophil behavior following drug treatment in *TgBAC(mpx:EGFP)* cryoinjured ventricles. (B) Representative maximum-intensity projection timelapse images of neutrophils at 24 h post-cryoinjury (hpci) in control, DEX- and LPS-treated ventricles, showing decreased and increased overall motility upon DEX and LPS treatment (magenta arrowheads; magenta lines), respectively. Frames are shown every 2 min from acquisitions taken every 10 s (see Movies 1 and 4). Yellow dashed rectangles outline the magnified areas shown in C. (C) Representative high magnification maximum-intensity projection images of neutrophils from B, highlighting decreased and increased cell shape changes in neutrophils from DEX- and LPS-treated hearts, respectively (see Movies 1 and 4). Four ventricles were imaged and analyzed for each condition. Scale bars: 10 µm.

Neutrophils are highly motile cells exhibiting high plasticity and rapidly adapting their behaviors to the microenvironment (Zhang et al., 2024). We first aimed to determine whether modulating the inflammatory activity of neutrophils was sufficient to induce behavioral changes. For this purpose, we cryoinjured cardiac ventricles from neutrophil-labeled *TgBAC(mpx:EGFP)* zebrafish to induce rapid neutrophil recruitment. Although this transgene is considered highly neutrophil-specific and is widely employed due to its strong expression (Bevan et al., 2020; Bruton et al., 2022; Lai et al., 2017), recent work has shown that a small subset of macrophages can express it in adult hearts (Wei et al., 2023). It is therefore possible that a minority of *mpx:*EGFP^+^ cells are in fact macrophages. Hearts were then explanted at 24 h post-cryoinjury (hpci), when many neutrophils populate the injured tissue (Goumenaki et al., 2024), and exposed to dexamethasone (DEX), an inflammation-suppressor drug (Coutinho and Chapman, 2011) or lipopolysaccharide (LPS), a pro-inflammatory molecule (Erridge et al., 2002; Page et al., 2022) (Fig. 1A), both routinely used in zebrafish (Xie et al., 2020; Toso et al., 2023). We then applied a fast-acquisition imaging approach, based on spinning disc microscopy at a high frame rate to capture fine time-resolved changes in cell morphology and location. To ensure consistency across treatment modalities, we focused on the injured tissue close to the border zone. In this way, we adapted an *ex vivo* system that can be used to screen drugs for their effect on immune cell behavior, while taking into account its limitations (e.g. lack of circulation and oxygenation, which can affect cell behavior especially in long-term cultures).

We observed that DEX induced a drastic reduction in neutrophil motility when compared with controls (Fig. 1B; Movie 1). Upon closer analysis, we observed that whereas neutrophils from control explants exhibited rapid changes in their morphology, indicative of highly dynamic behavior, neutrophils from DEX-treated ventricles displayed largely reduced morphodynamics (Fig. 1C; Movie 1). In other zebrafish experimental paradigms, such as in the larval fin amputation model, DEX has been shown to impair neutrophil motility, resulting in defective neutrophil recruitment (Xie et al., 2020) as well as aberrant neutrophil migration (Sharif et al., 2015). In the adult zebrafish heart, a previous study reported a reduction in the migratory activity of neutrophils following cryoinjury in *breakdance* mutants upon DEX treatment (Xu et al., 2019), which is consistent with our observations of the disruption in overall neutrophil kinetics. Moreover, DEX has also been shown to inhibit regeneration of the neonatal pig heart following myocardial infarction (Tao et al., 2020), as well as of the adult zebrafish heart following cryoinjury (Sallin and Jaźwińska, 2016), in line with the role of neutrophils in triggering an acute inflammatory wave in diverse regenerative contexts (de Preux Charles et al., 2016).

In contrast, neutrophil motility strongly increased upon LPS treatment when compared with controls (Fig. 1B; Movie 1), associated with pronounced morphodynamic behaviors (Fig. 1C; Movie 1). Although our analysis only included cells tracked for a minimum of 20 consecutive frames, potentially leading to a slight underrepresentation of the fastest-moving neutrophils in LPS-treated ventricles, the magnitude of LPS-induced behavioral changes was strong enough to remain clearly evident, even with this conservative filtering. LPS has been shown to increase neutrophil motility in a number of organs including the mammalian blood–brain barrier and lungs (Kim et al., 2020; Jeong et al., 2022), as well as in *in vitro* models (Boribong et al., 2019), where both neutrophil velocity and displacement were affected. In zebrafish, LPS has been shown to induce increased neutrophil infiltration into several organs (Yang et al., 2014; Zhang et al., 2016), coinciding with increased motility behavior.

Given that these experiments relied on 3-h incubation periods, we next asked whether incubation itself, even in control conditions, influences neutrophil behavior. We compared neutrophils from 24 hpci ventricles incubated for 3 h in control medium with those from freshly extracted 24 hpci ventricles. This analysis revealed a moderate increase in motility and morphodynamic behaviors in neutrophils from incubated ventricles compared with freshly extracted ones (Fig. S1, Movie 2, Table S4). Although modest when compared with the robust effects induced by LPS and DEX, and not affecting the conclusions drawn from our drug treatment assays, this basal effect should nonetheless be taken into account when using this setup to assess the immunomodulatory impact of drugs on neutrophil behavior. To test whether neutrophil behaviors were being influenced by potential incubation-induced apoptosis, we incubated cryoinjured hearts in control medium for 5 h, substantially longer than for the drug treatment experiments, and observed that neutrophils remained dynamically active with no morphological signs of apoptosis (Movie 3).

Altogether, our data reveal a direct association between the inflammatory activity of neutrophils and their motility and morphodynamic behaviors in the cryoinjured zebrafish heart. They also illustrate the utility of an adult organ that can be used to track the behavioral repertoire of neutrophils in more detail and at higher resolution.

### Neutrophils segregate into behaviorally distinct subsets based on their inflammatory state

**Fig. 2.**
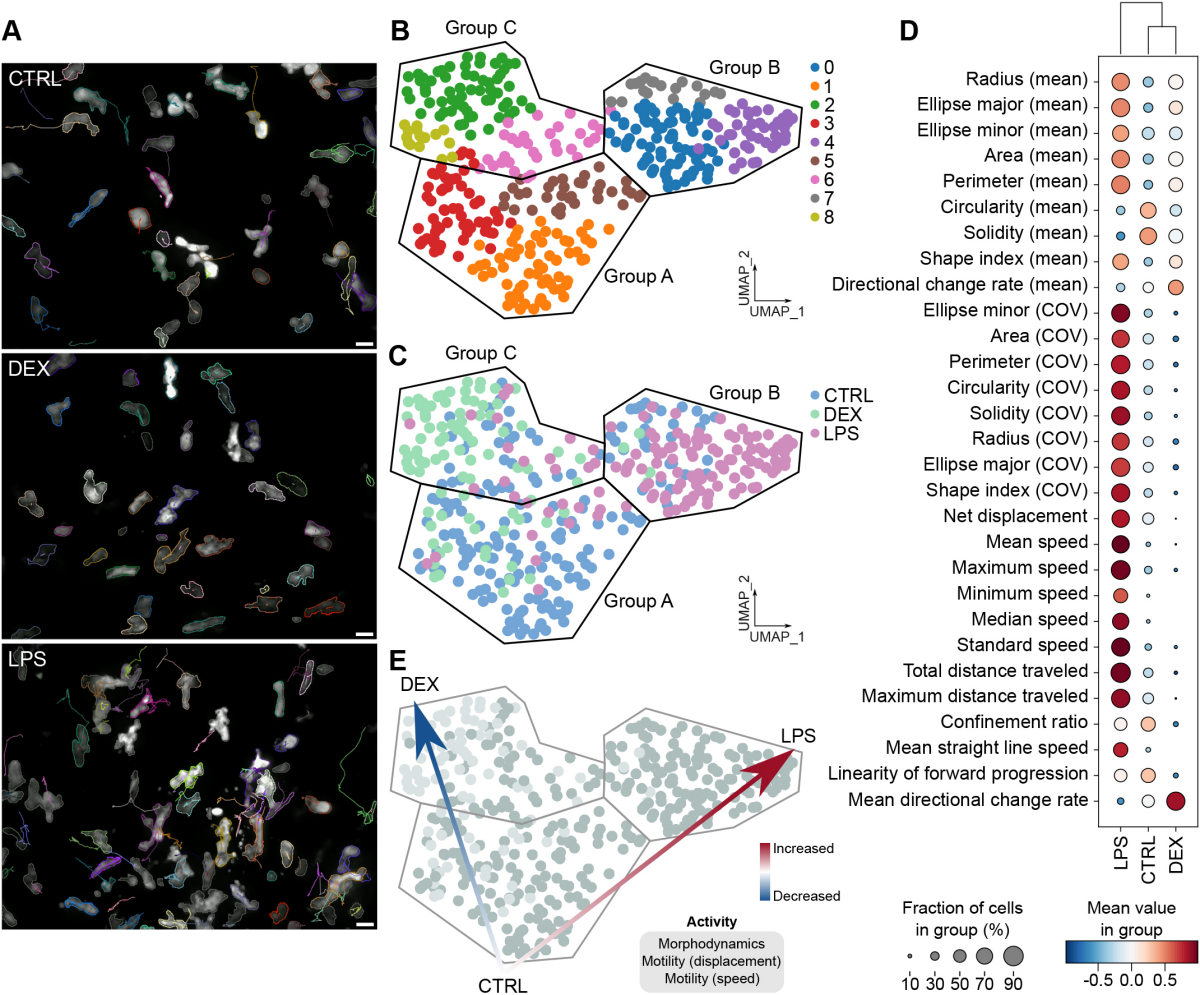
**Neutrophils segregate into behaviorally distinct subsets in response to immunomodulators.** (A) Representative images from control, DEX-treated and LPS-treated cryoinjured ventricles showing neutrophil segmentation and tracks (colored lines) using the TrackMate-Cellpose plugin (see Movie 4). (B) UMAP representation of nine distinct neutrophil clusters based on 29 distinct motility and morphological parameters from all control, DEX-treated and LPS-treated ventricles combined. Clusters are organized into three major groups (i.e. group A, group B and group C) according to hierarchical clustering (see Fig. S2). Data used for the analysis can be found in Table S5. (C) UMAP representation showing the distribution of neutrophils by treatment, showing the predominance of control neutrophils in group A, of neutrophils from DEX-treated hearts in group C, and of neutrophils from LPS-treated hearts in group B. (D) Dot plot illustrating the enrichment (i.e. mean value and percentage of neutrophils) of the various parameters across treatment groups, showing decreased and increased enrichment in morphodynamics and motility (i.e. speed and displacement) in neutrophils from DEX- and LPS-treated samples, respectively. Dendrogram at the top indicates hierarchical clustering of the groups. (E) UMAP representation summarizing treatment-dependent clustering organization according to overall activity as assessed by morphodynamics and motility. Analyses were carried out on neutrophils (*n*=177 for CTRL, 89 for DEX and 127 for LPS) from four combined ventricles per group.

To further dissect the behavioral repertoire of neutrophils, we performed a more comprehensive parametric analysis with DEX- and LPS-treated cardiac ventricles, as well as corresponding controls (Fig. 2). For this purpose, we segmented and tracked individual neutrophils (Fig. 2A; Movie 4) and measured 21 motility (i.e. speed and displacement) and morphological parameters. Although motility parameters directly reflect the corresponding behaviors by integrating data over time (e.g. speed and distance traveled), morphological parameters are discrete measurements (e.g. area and perimeter) captured at distinct timepoints during image acquisition. Therefore, for each morphological parameter, apart from measuring the mean values across all timepoints during image acquisition, we also calculated the coefficient of variation (COV). This analysis resulted in a total of 29 parameters extracted from the tracking of individual neutrophils (Fig. 2; Fig. S2, Table S5), which were used for dimensional reduction and rendering of a 2D UMAP representation of cell behavior.

Cell segmentation and clustering of behaviorally distinct immune cells [e.g. natural killer (NK) cells] has been performed *in vitro,* revealing novel aspects of immune cell behaviors (Shannon et al., 2024). Here, we identified nine behaviorally distinct neutrophil subsets classified as clusters 0 to 8 (Fig. 2B), which organized into three major groups (Fig. 2B; Fig. S2). Interestingly, apart from residual overlap, each group is a clear representation of treatment-dependent behavioral states, with group A primarily composed of neutrophils from control hearts, and groups B and C predominantly composed of neutrophils from LPS- and DEX-treated hearts, respectively (Fig. 2C). Interestingly, the distribution of neutrophils into distinct clusters within each treatment group, even in control conditions, highlights a clear heterogeneity in neutrophil behaviors. We then aimed to determine the parameters that influence the segregation of the various clusters (Fig. S2) across the different treatments. This study, which aims to capture global behavioral profiles at the cell level while resolving heterogeneity, treats behavior as a cell-intrinsic property and therefore considers individual neutrophils as distinct biological entities. However, complementary per-ventricle single-parameter statistics are also provided (Tables S1–S3). Consistent with our previous observations, speed parameters were markedly increased for neutrophils in LPS-treated hearts and, conversely decreased for neutrophils in DEX-treated hearts, when compared with controls (Fig. 2D; Fig. S3A–D). Similarly, most displacement parameters changed upon LPS and DEX treatment (Fig. 2D; Fig. S3E–K), with total distance and net displacement strongly increased by LPS and reduced by DEX (Fig. 2D; Fig. S3I,J). Among the neutrophils from LPS-treated samples, these changes were mostly associated with cluster 4, the most behaviorally distinct from those of other treatment groups (Fig. S2). Although DEX strongly arrested overall neutrophil motility, our data reveal an increase in directional change rate associated with decreased confinement ratio and linearity of forward progression (Fig. 2D; Fig. S3G,H,K). This pattern is indicative of quick and random directional changes within small movements (i.e. oscillatory behavior) in a confined area, consistent with impaired chemotactic navigation (Byrne et al., 2014). A combination of morphological and motility information has recently been shown to predict functional macrophage phenotypes, including their anti- and pro-inflammatory subsets (Kesapragada et al., 2024). In our study, along with the described motility parameters, a number of morphological features, which are further discussed in the following section, also underlie the segregation of neutrophils into LPS- and DEX-specific clusters.

Together, our data show that immunomodulation induces behavioral segregation of neutrophils, suggesting that their dynamic behavior can be predictive of inflammatory activity (Fig. 2E).

### Cell shape variability complements morphological indicators of neutrophil activity

**Fig. 3.**
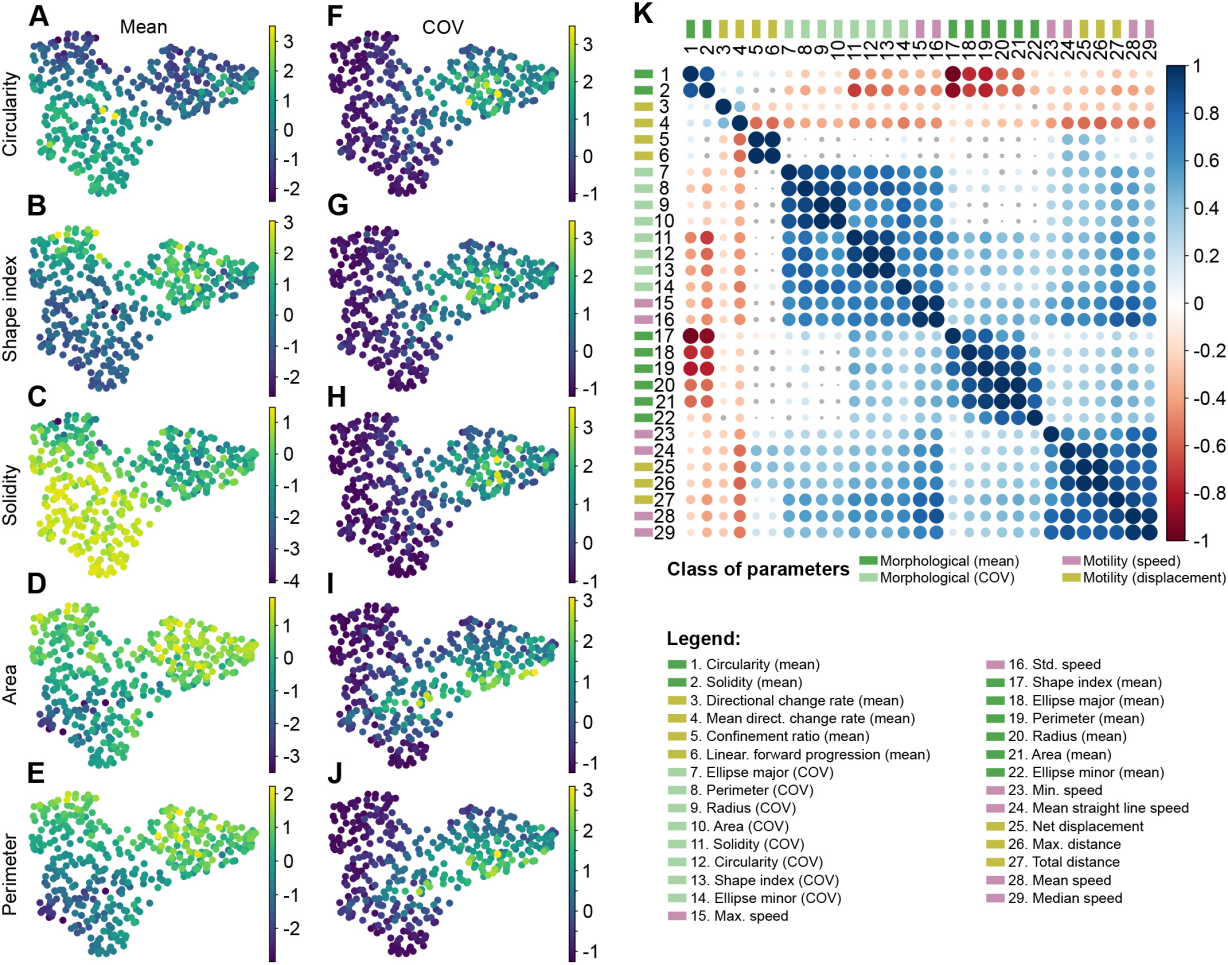
**Cell shape variability complements morphological indicators of neutrophil activity.** (A–J) UMAP representation of the enrichment of mean values (A–E) and corresponding coefficients of variation (COVs; F–J) of representative morphology-related parameters in neutrophils from all treatment groups. Although mean values of parameters vary considerably between conditions, COVs are exclusively enriched in neutrophils from group B (i.e. mostly composed of neutrophils from LPS-treated samples), highlighting that cell shape variability, and not discrete morphological assessments alone, is an accurate proxy of morphodynamics and neutrophil activity. High-to-low enrichment values are depicted by a yellow-to-blue color scale, respectively. (K) Correlogram showing pairwise correlations among all morphology-related and motility parameters across conditions. Strong positive correlations (dark blue) are observed between parameters within the same behavioral classes (i.e. motility or morphology-related), but also between different classes, particularly between motility and morphological COV. Positive and negative correlations are shown in blue and red, respectively; dot size represents Pearson's correlation coefficient (gray dots show non-significant correlations; *P*<0.05). Analyses included neutrophils (*n*=177 for CTRL, 89 for DEX and 127 for LPS) from four combined ventricles per group.

Neutrophils alter their shape in response to stimuli, including chemotactic signals (Ehrengruber et al., 1996). It is generally accepted that resting neutrophils are spherical and exhibit a smooth surface, whereas activated neutrophils spread out, acquire morphological polarity, form lamellipodia and pseudopodia, and undergo membrane ruffling (Ehrengruber et al., 1996). Here, we observed a decrease in mean circularity (Fig. 2C, Fig. 3A; Fig. S4A) and an increase in mean shape index (Fig. 2C, Fig. 3B; Fig. S4B) upon LPS treatment, suggestive of a non-spherical morphology, as well as a decrease in mean solidity (Fig. 2C, Fig. 3C; Fig. S4C), which indicates membrane ruffling and pseudopodia formation. Altogether, these data are consistent with increased neutrophil activation. In addition, LPS induced a marked increase in mean dimension-related parameters (e.g. area and perimeter), as observed in the 2D plane (Fig. 2C, Fig. 3D,E; Fig. S4D,E), likely in line with an increased pro-inflammatory profile. In fact, increased neutrophil size and volume have been reported in inflammatory conditions such as in lung cancer (Rutkowska et al., 2024) and inflammatory bowel disease (Aydemir et al., 2017). Neutrophil volume has also been shown to increase following myocardial infarction and to positively correlate with infarct size in pigs (van Hout et al., 2015).

In our study, LPS-induced changes in variability (i.e. COV) of all aforementioned morphological parameters were more pronounced than changes in their mean values (Fig. 2C, Fig. 3F–J; Fig. S4F–J). Moreover, neutrophils from DEX-treated hearts displayed similar, although milder, alterations in mean morphological parameters (i.e. circularity, shape index, solidity, area and perimeter) when compared with those from LPS-treated hearts (Fig. 2C, Fig. 3A–E; Fig. S4A–E), possibly in line with the above-mentioned oscillatory behavior. However, as opposed to LPS-treated conditions, the COV of these parameters was drastically reduced upon DEX treatment compared with controls (Fig. 2C, Fig. 3F–J; Fig. S4F–J). Together, these data indicate that morphological variability over a given time period, rather than discrete morphological snapshots alone (Fig. S4K–M), more directly reflects morphodynamics and the activation state of neutrophils.

We then generated a correlogram to identify links between the 29 measured parameters, particularly between motility and morphology (Fig. 3K). As expected, strong correlations were observed within the same class of parameters. For example, apart from distance-related parameters, which only mildly correlated with directionality-related ones, displacement parameters displayed strong correlations among themselves as well as with speed parameters. Similarly, strong correlations were observed among morphological parameters. More importantly, we observed correlations between most motility (i.e. displacement- and speed-related) and morphological metrics, further highlighting the link between motility and morphological behaviors. Notably, these correlations were stronger for morphological COVs than for their counterpart mean values, emphasizing the value of analyzing morphological variability as a readout for morphodynamics and neutrophil activity.

### Neutrophils exhibit heterogeneous but temporally enriched behavioral states during zebrafish cardiac regeneration

**Fig. 4.**
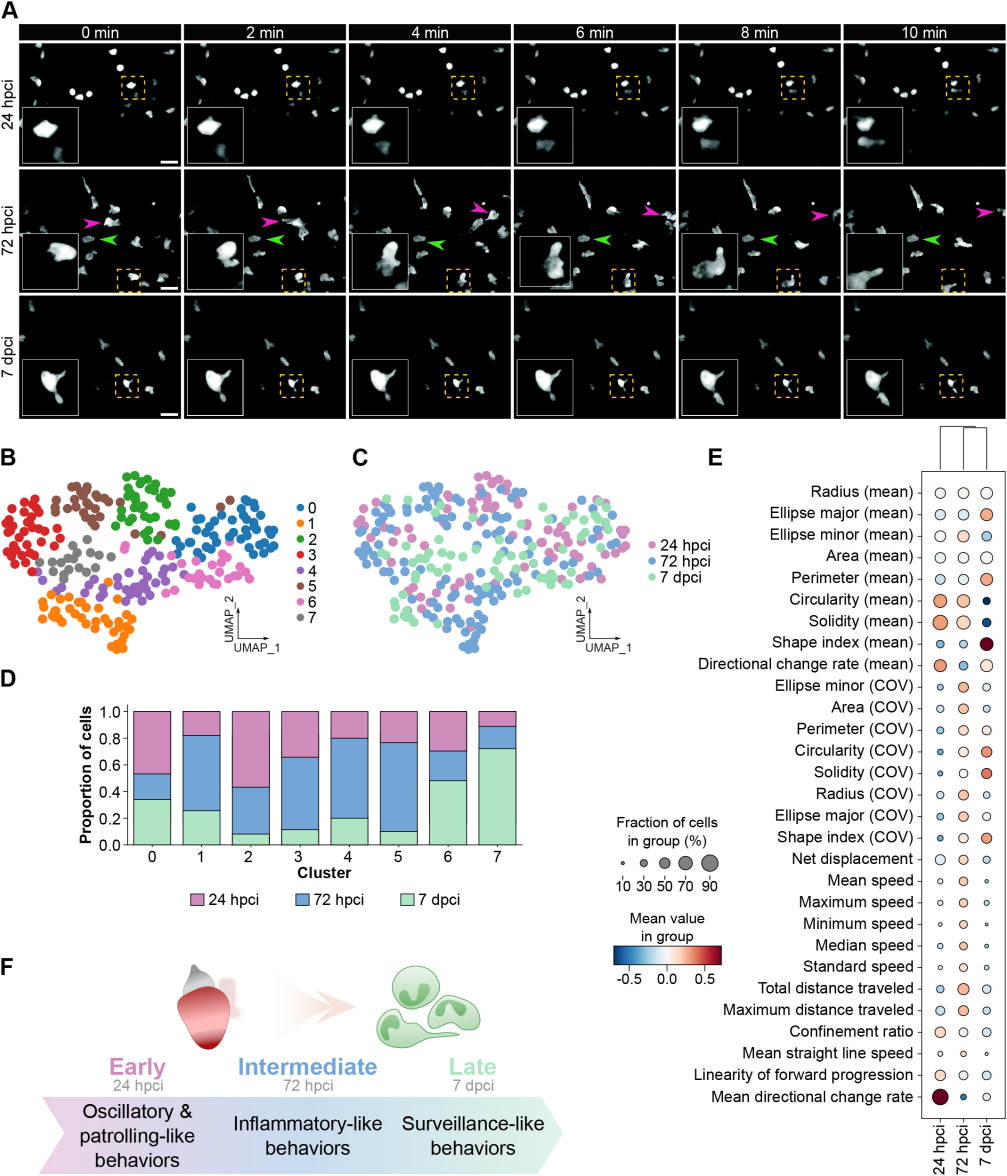
**Neutrophil behavior is heterogeneous and changes during the regenerative process.** (A) Representative maximum-intensity projection timelapse images of neutrophils from *TgBAC(mpx:GFP)* cryoinjured ventricles at 24 and 72 hpci and 7 dpci, showing morphological and motility differences between regeneration stages, including overall increased but heterogeneous behavioral dynamics at 72 hpci, with highly motile (magenta arrowheads) and poorly motile or immotile (green arrowheads) neutrophils. Frames are shown every 2 min from acquisitions taken every 10 s (see Movies 5 and 6). Yellow dashed rectangles outline areas magnified in the insets. (B) UMAP representation of eight distinct neutrophil clusters based on 29 behavioral parameters from all ventricles from the three regeneration timepoints combined. Data used for the analysis can be found in Table S6. (C) UMAP representation showing the distribution of neutrophils by timepoint, documenting behavioral heterogeneity. (D) Segmented bar graphs showing the proportion of neutrophils from each timepoint within each cluster. Chi-square test (*P*<0.0001). (E) Dot plot illustrating the enrichment (i.e. mean value and percentage of neutrophils) of the various parameters across regeneration timepoints. Dendrogram at the top indicates hierarchical clustering of the groups. (F) Schematic illustration summarizing the predominant behavioral states of neutrophils across different stages of cardiac regeneration, based on parametric analyses. Analyses were carried out on neutrophils (*n*=86 for 24 hpci, 113 for 72 hpci and 69 for 7 dpci) from combined ventricles (4, 4 and 5 ventricles for 24 hpci, 72 hpci, and 7 dpci, respectively) per group. Scale bars: 10 µm.

Neutrophils are among the first immune responders to cardiac injury (Ma, 2021), including in the regenerating zebrafish heart (Bevan et al., 2020). Whereas neutrophils are required for the regeneration process by orchestrating an acute inflammatory wave, their activity needs to be tightly regulated to avoid deleterious effects on the regenerative response (Xu et al., 2019). However, how neutrophil behaviors (i.e. motility and morphodynamics) change during regeneration, and how they potentially relate with their inflammatory activity is underexplored. So far, we have focused on neutrophils from hearts collected at 24 hpci. Here, we analyzed and integrated data of neutrophils from hearts extracted at 24 hpci (i.e. during the peak of neutrophil accumulation), 72 hpci (i.e. intermediate stage of neutrophil accumulation) and 7 dpci (i.e. late stage of neutrophil accumulation) (Lai et al., 2017; Xu et al., 2018; Bevan et al., 2020; Ryan et al., 2020). In an initial analysis (Fig. 4A; Movie 5), a majority of neutrophils at 24 hpci exhibited low motility and appeared more rounded compared with neutrophils at other timepoints. In contrast, neutrophils at 72 hpci displayed motility heterogeneity (i.e. mostly highly motile cells, with a few immotile ones) accompanied by a more elongated shape with pronounced morphological changes, including protrusion retraction cycles (Fig. 4A; Movie 5). At 7 dpci, neutrophils displayed highly irregular shapes accompanied by low motility (Fig. 4A; Movie 5). As above, following cell segmentation (Movie 6) and analysis, we performed dimensional reduction and rendered UMAPs to better understand the neutrophil behavioral repertoire during cardiac regeneration (Fig. 4B), based on the same 29 parameters (Table S6). Although the identified clusters do not fully segregate by timepoint (Fig. 4C), due to parametric variability (Fig. S5), their distribution reveals distinct enrichment patterns – clusters 0 and 2 are enriched for neutrophils from 24 hpci, clusters 1, 3, 4 and 5 for neutrophils from 72 hpci, and clusters 6 and 7 for neutrophils from 7 dpci (Fig. 4D). This analysis further confirms neutrophil behavioral heterogeneity which, although more pronounced at 72 hpci, is present throughout cardiac regeneration. Moreover, the transgenic line used in this study is not specific for activated neutrophils, therefore neutrophils at several activation stages, including immature neutrophils, were part of the analysis potentially adding to the observed behavioral heterogeneity.

In comparison with the other timepoints, only a small subset of neutrophils is actively motile at 24 hpci (Fig. S6). At this timepoint, neutrophils exhibit increased mean directional change rate (Fig. 4E; Fig. S5F, Fig. S7), indicating reduced directionality and increased turning. This motility pattern, with the neutrophils exhibiting various confinement ratios (Fig. 5E; Fig. S5I), is consistent with oscillatory and patrolling-like behaviors (Pizzagalli et al., 2019) at this early timepoint. Interestingly, neutrophils at 72 hpci include more actively motile cells (Fig. S6), with increased speed and displacement accompanied by increased morphodynamics, as assessed by the COV of morphological parameters, especially when compared with neutrophils at 24 hpci (Fig. 4E; Fig. S5O–S). Overall, neutrophils at 72 hpci exhibit a behavior that is relatively more similar to the pro-inflammatory state induced by LPS (Fig. 2C). Neutrophil inflammatory activity is thought to peak prior to 72 hpci, as neutrophil numbers peak at 24 and 48 hpci within the injured cardiac tissue (Ryan et al., 2020; Feng et al., 2025). However, whether neutrophil numbers correlate with their inflammatory activity is not clear. In addition, a study focusing on the zebrafish cardiac amputation model has identified transcriptionally distinct neutrophil subsets 1 day post-amputation (Peterson et al., 2024). These neutrophils display variable expression of inflammation-related genes, indicating transcriptional heterogeneity and variability in their inflammatory profile (Peterson et al., 2024). Similarly, distinct neutrophil subsets have also been identified in the cardiac cryoinjury model (Wei et al., 2023). Hence, it is possible that although neutrophil numbers decrease by 72 hpci, at least some of them exhibit a heightened inflammatory activity, which is also represented by their behavioral profile. We also consistently observed phagocytic-like behaviors at 72 hpci, not often observed in the other timepoints. As the *mpx:EGFP* transgenic line used in this study can also label some macrophage populations, as mentioned above, it is possible that these phagocytic-like behaviors are being displayed by macrophages and not neutrophils. Altogether, these data suggest that neutrophil numbers alone are not sufficient to infer their inflammatory state and point to an intrinsic neutrophil inflammatory spectrum that warrants further investigation.

Neutrophils at 7 dpci include a small subset of actively motile cells as also observed at 24 hpci (Fig. S6), and they also exhibit overall decreased mean circularity and solidity and increased mean shape index (Fig. 4E; Fig. S5J,L, Fig. S7). These data are consistent with our initial observations on neutrophils exhibiting non-spherical and irregular shapes with extending protrusions. Some neutrophils, especially those from cluster 7, exhibit increased COV of these parameters (Fig. S7), indicating some degree of morphodynamics. However, when compared with those at 72 hpci, neutrophils at 7 dpci exhibit reduced overall motility. Apart from increased morphodynamics and distinct morphological features, and despite the intrinsic variability within timepoints, the overall behavior of neutrophils at 7 dpci resembles more that of neutrophils at 24 hpci. Altogether these data are suggestive of a more ‘arrested’ (Pizzagalli et al., 2019) and more surveillance-like (i.e. sentinel) behavior, with neutrophils also potentially engaging in interactions with other cells and the extracellular matrix. The irregular shape of neutrophils at 7 dpci, associated with highly decreased motility, further reinforces our conclusion that morphology alone is not indicative of overall dynamics.

Altogether, our data reveal marked neutrophil behavioral diversity throughout the cardiac regenerative process, indicating that neutrophils do not behave as single homogeneous populations fixed to timepoints, but instead occupy overlapping behavioral states whose proportions shift over time. This diversity underscores the presence of a broader spectrum of neutrophil states than is typically appreciated. We propose that, during regeneration, these states might be enriched in oscillatory and patrolling-like behaviors at early neutrophil accumulation phases, inflammatory-like behaviors at intermediate phases, and surveillance-like behaviors at late phases (Fig. 4F). These interpretations provide a basis for further investigation of the dynamic roles of neutrophils during regeneration.

Overall, this study introduces a methodological platform to analyze immune cell behavior, enabling robust assessment of the immunomodulatory properties of different compounds. It also provides an analytical framework to further investigate the inflammatory behaviors that underlie regenerative processes, thereby complementing the study of inflammatory conditions.

## MATERIALS AND METHODS

### Zebrafish handling and husbandry

Zebrafish larvae were raised under standard conditions. Adult fish were maintained in 3.5 l tanks at a stock density of ten fish per liter with the following parameters: water temperature, 27–27.5°C; light–dark cycle, 14 h:10 h; pH, 7.0–7.5; conductivity, 750–800 µS cm^−1^. Zebrafish were fed three to five times a day, depending on age, with ZEBRAFEED (Sparos, Portugal) and live food (*Artemia salina*). Health monitoring was performed twice a year. All procedures performed on animals conformed to the guidelines from Directive 2010/63/EU of the European Parliament on the protection of animals used for scientific purposes and were approved by the Animal Protection Committee (Tierschutzkommission) of the Regierungspräsidium Darmstadt (ref. no. B2/1229). All efforts were made to minimize pain, distress and discomfort. Anesthesia was performed by incubating zebrafish in 0.6mM tricaine solution (MS-222; Sigma-Aldrich, MO, USA). Euthanasia was performed using a lethal dose of anesthetic, prior to heart collection.

### Zebrafish lines and cardiac cryoinjury

*TgBAC(mpx:GFP)^i114^* transgenic zebrafish (3 to 12 months of age) were used in this study (Renshaw et al., 2006). Cardiac cryoinjury was performed in adult zebrafish hearts as described previously (Chablais et al., 2011; González-Rosa et al., 2011; Schnabel et al., 2011; Cardeira-da-Silva et al., 2024). Zebrafish were briefly anesthetized with tricaine and transferred onto a wet sponge with their ventral side up. A small incision was made in the thoracic region exposing the heart ventricle, and a cryoprobe precooled in liquid nitrogen was applied to the ventricular apex until thawing. Cryoinjured fish were transferred into fresh system water and left to recover. For each timepoint or drug treatment condition four fish of mixed genders were analyzed.

### Heart collection, explant handling, and drug treatment

Cryoinjured hearts were collected at the required timepoints and transferred to Dulbecco's modified Eagle's medium (DMEM; Thermo Fisher Scientific, USA) solution. For drug treatments, collected hearts were incubated either in DMEM (0.2% DMSO; control), DMEM with 125 μM DEX (0.2% DMSO), or DMEM with 25 μg/ml LPS for 3 h at 28°C. DEX and LPS were sourced from Sigma-Aldrich. Explants were then transferred into a 25% tricaine (0.4%)/75% DMEM solution until heartbeat cessation. The hearts were then transferred to a one-well glass-bottom imaging chamber (35 mm petri dish, 10 mm microwell, P35G-1.5-10-C, MatTek Corporation, MA, USA) with a transfer pipet, and kept immersed in the same tricaine/DMEM solution during image acquisition. For regeneration studies without drug treatments, hearts were stopped immediately following collection as described and imaged directly.

### Imaging

Live imaging of explanted hearts was performed using a Cell Observer spinning disk microscope (Zeiss, Germany) with a CSU-X1 scanner unit (Yokogawa, Japan) and ORCA flash4.0 sCMOS camera (Hamamatsu Photonics, Japan), at 24°C. Movies of neutrophils were acquired by first imaging the heart using the 10×/0.3D air objective. Once the injured region was identified, timelapse videos of the border zone region were acquired using a 40×/1.1 water immersion lens. For each heart a single border-zone region was imaged. Time series of entire *z*-stacks of border-zone regions were acquired with 60 frames at 6.66 frames/s, at 10 s intervals. Timelapse videos were exported to ImageJ v1.52k (Wayne Rasband, National Institutes of Health, Bethesda, MD, USA) for further processing.

### Image processing, cell segmentation and tracking

Pre-processing and tracking were performed in ImageJ using a TrackMate-Cellpose plugin (Stringer et al., 2021; Ershov et al., 2022; Pachitariu and Stringer, 2022). The data was normalized by two consecutive bleach corrections based on histogram matching and simple ratio to enhance the visibility of cellular features. We trained a custom Cellpose 2.0 model by finetuning the published Cyto2 Model with frames 1, 30 and 59 (beginning, middle and end of the timelapse to ensure a stable tracking throughout the normalized video; Pachitariu and Stringer, 2022). We then used this custom model through the TrackMate–Cellpose plugin to segment the cells. Cells were then tracked with a simple LAP tracker allowing 15 µm linking distance and a maximum frame gap of 2. To ensure high-quality trajectories, only cells tracked for at least 20 consecutive frames were retained, excluding truncated tracks when cells transiently leave the focal plane or field of view. The tracking values were exported .csv files for further analysis.

### Data analysis, visualization and statistics

To analyze the tracked data, we selected 21 spot, edge and track parameters, which were then used to create a data matrix with all cells and these parameters. The data matrix was processed in Scanpy, a popular Python library for single-cell analysis (Wolf et al., 2018). We normalized the data by applying the log1p transformation, extracted highly variable parameters, and scaled the values between −5 and 5. In parallel, we calculated the coefficient of variation (COV) during image acquisition for the eight morphological parameters tracked (i.e. area, perimeter, circularity, solidity, radius, shape index, ellipse major and ellipse minor), resulting in a total of 29 parameters used in further analyses.

We then performed a dimensional reduction using principal component analysis (PCA), calculated the neighbors and embedded the data into a two-dimensional space using uniform manifold approximation and projection (UMAP). Clustering was performed using the Leiden algorithm, and visualized using a variety of tools, including UMAP and Scanpy's built-in visualization functions. Data used for the analysis can be found in Tables S5 and S6. The processed data are available through the CellxGene platform. Dot plots were generated using the Scanpy package with default settings (Wolf et al., 2018).

The percentages of poorly motile or immotile (<10 µm) and highly motile (>10 µm) cells were calculated based on the total distance traveled.

Statistical comparisons between groups were performed with GraphPad Prism (v.9). Data that were normally distributed according to the Shapiro–Wilk test were further analyzed with the one-way ANOVA test followed by post hoc Tukey's test for multiple comparisons. The non-parametric Kruskal–Wallis test was applied for global per-ventricle comparisons across groups. A chi-square test was applied to test categorical variables, using the observed frequency values. The significance level was set to 0.05 for all tests.

To study linear relationships between pairs of parameters, correlation matrices were generated by calculating Person's correlation coefficients. Calculations were performed and correlogram generated using RStudio version 2024.12.0+467 (https://www.r-project.org/), and the *Hmisc* (version 5.2-3; https://cran.r-project.org/web/packages/Hmisc/index.html) and *corrplot* (version 0.95; https://cran.r-project.org/web/packages/corrplot/index.html) packages. The significance level was set to 0.05 for all bivariate correlations.

## Supplementary Material

10.1242/joces.264418_sup1 Supplementary information

Table S4. Complete set of data of all analyzed neutrophils from freshly collected cryoinjured ventricles and cryoinjured ventricles following 3 h of incubation in control medium.

Table S5. Complete set of data of all analyzed neutrophils from explanted cryoinjured ventricles upon incubation in control medium (CTRL), DEX or LPS.

Table S6. Complete set of data of all analyzed neutrophils from cryoinjured ventricles collected at 24 hpci, 72 hpci and 7 dpci.
