# Supplementary material for: Ex vivo imaging reveals neutrophil behaviors in the adult zebrafish heart
Source: J Cell Sci. 2026 May 11;139(9):jcs264418. doi: 10.1242/jcs.264418 (PMC13245898; doi:10.1242/jcs.264418)
Supplement: Supplementary information [file joces-139-264418-s1.pdf]

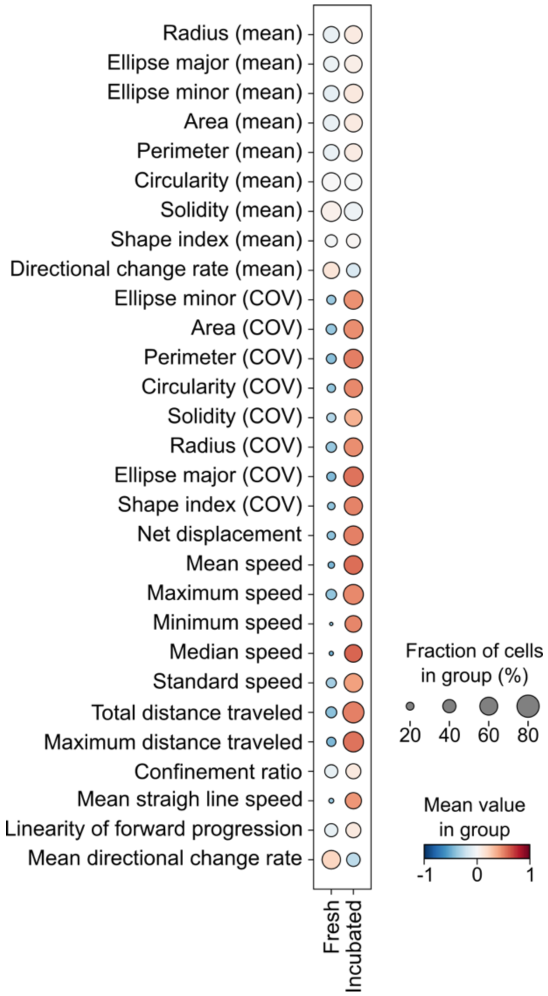

Fig. S1. Incubation of explanted hearts activates neutrophil motility and morphodynamic behaviors. Dot plot illustrating the enrichment (i.e., mean value and percentage of neutrophils) of the various parameters in cryoinjured cardiac ventricles incubated for 3 hours in control medium compared with freshly collected cryoinjured ventricles. Data include all neutrophils (*N* = 185 for Fresh, 152 for Incubated) from combined ventricles per group (5 for Fresh, 6 for Incubated).

Journal of Cell Science • Supplementary information

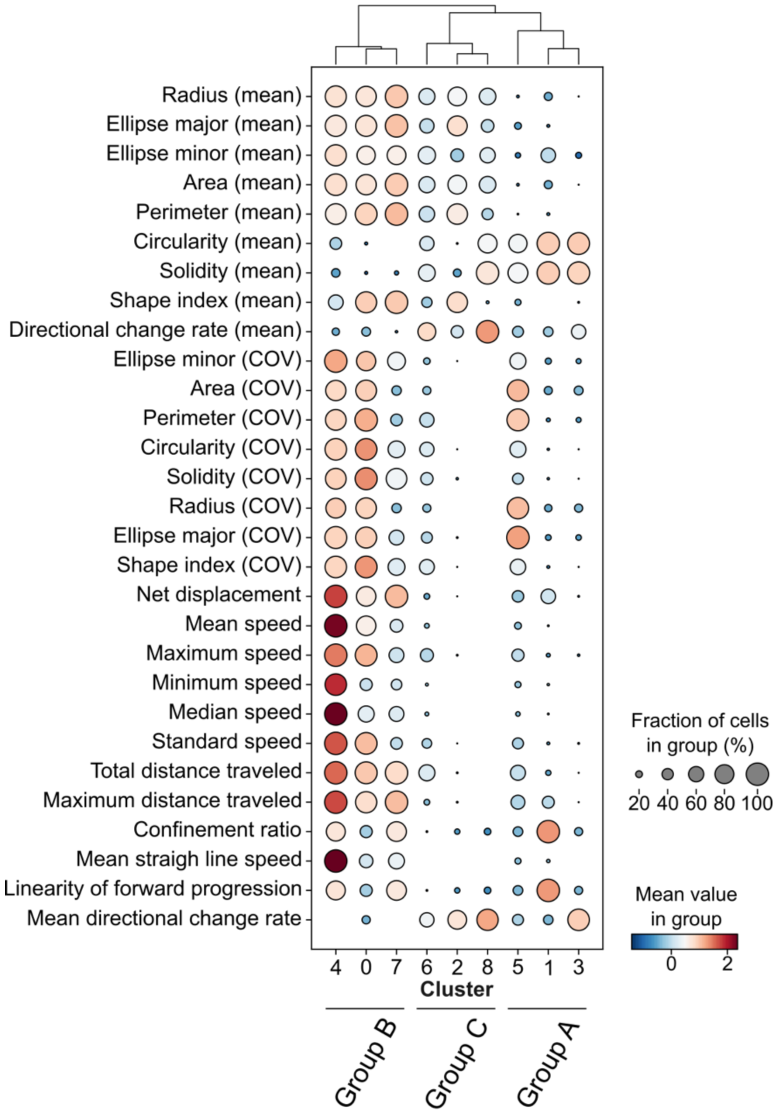

Fig. S2. Neutrophils segregate into distinct behavioral clusters. Dot plot illustrating the enrichment (i.e., mean value and percentage of neutrophils) of the various parameters across clusters. The analysis includes all neutrophils from all control, DEX-treated, and LPS-treated cryoinjured ventricles combined. Clusters are sub grouped according to hierarchical clustering, with group B, especially cluster 4, exhibiting the most pronounced differences. Data include all neutrophils (*N* = 177 for CTRL, 89 for DEX, and 127 for LPS) from 4 combined ventricles per group.

Journal of Cell Science • Supplementary information

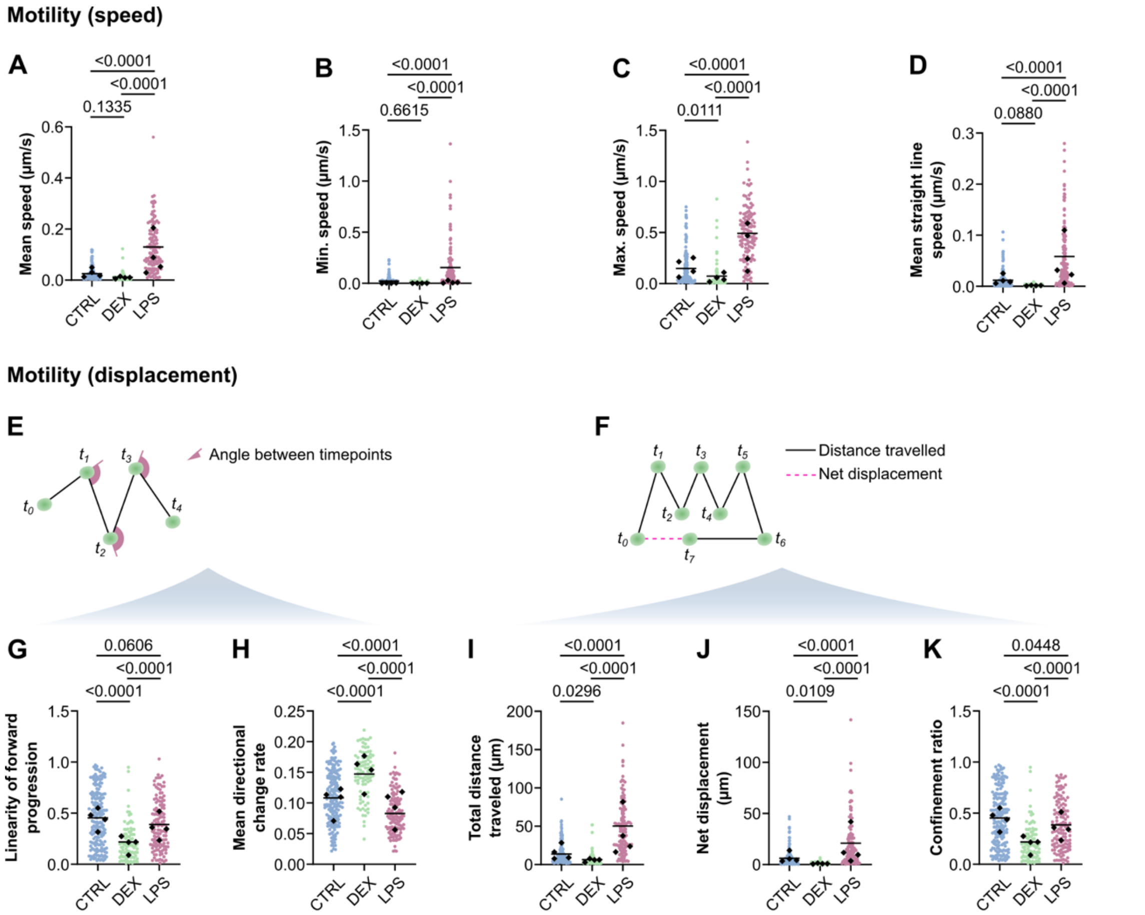

Fig. S3. Immunomodulation alters neutrophil motility. (**A-D**) Beeswarm plots showing reduced and strongly increased speed-related motility parameters in neutrophils upon DEX and LPS treatments, respectively, compared with control (CTRL). (**E**, **F**) Schematic representations of simplified motility patterns, highlighting angular movements (E), as well as distance travelled *versus* net displacement (F). The different types of displacement, and their integration, depend on the combination of the different parameters below. (**G-K**) Beeswarm plots showing displacement-related motility parameters, highlighting loss of directionality (G, H) in neutrophils from DEX-treated cryoinjured ventricles, as well as reduced and increased displacement (I, J, K) in neutrophils from DEX- and LPS-treated cryoinjured ventricles, respectively. In all graphs, colored dots represent individual neutrophils (*N* = 177 for CTRL, 89 for DEX, and 127 for LPS) from 4 combined ventricles per group; the black horizontal bars represent the corresponding mean. Black diamonds represent mean values per ventricle. One-way ANOVA and Tukey's post hoc test for multiple comparisons of individual neutrophils across treatments (*P* values included in the graphs). Complementary per-ventricle statistics are provided in Table S1.

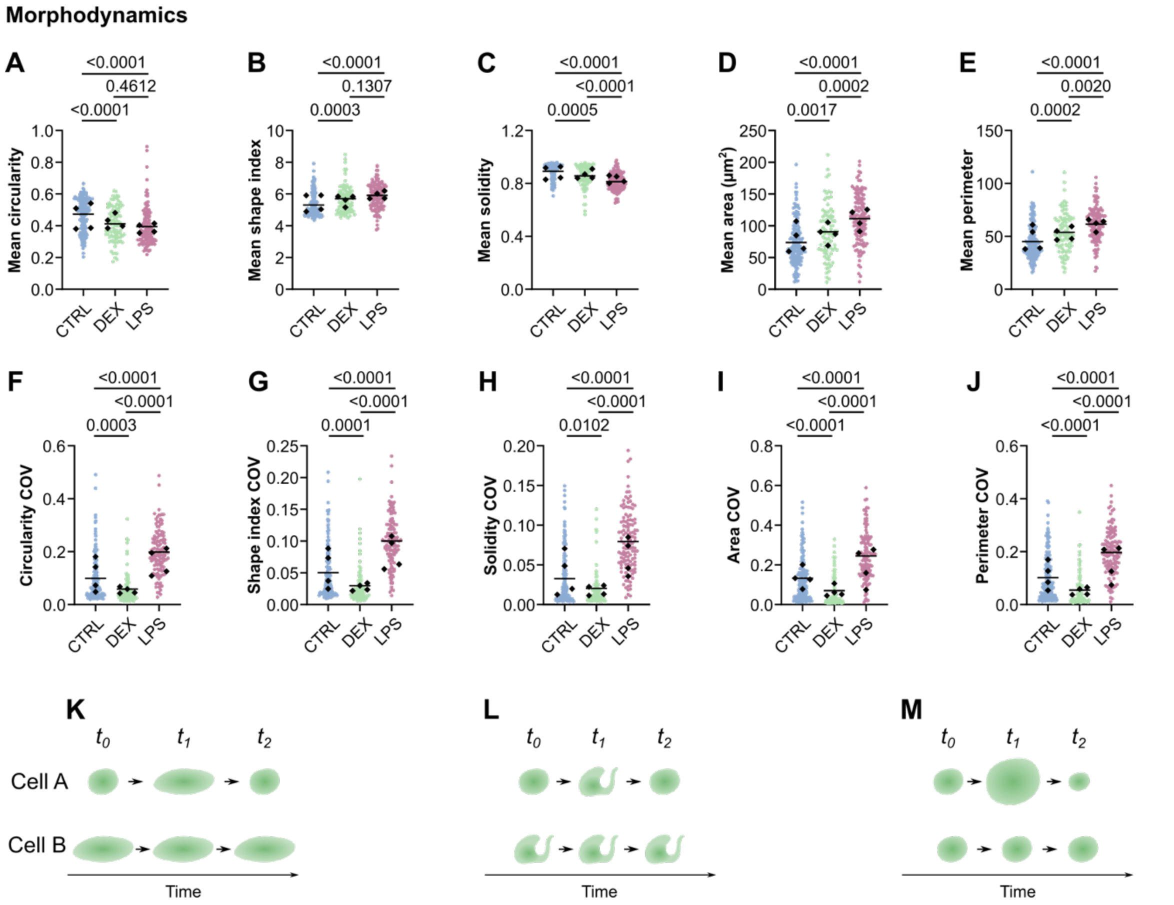

Fig. S4. Immunomodulation alters neutrophil morphodynamics. (A-J) Beeswarm plots showing morphodynamic parameters in neutrophils from DEX- and LPS-treated cryoinjured ventricles compared with control (CTRL). While similar changes in mean morphological values (A-E) are induced by both DEX (to a lesser extent) and LPS, the corresponding COVs (F-J) drastically change in opposite directions upon treatment (i.e., increased in DEX and decreased in LPS). (**K-M**) Schematic illustrations depicting the temporal variation of several cell shape features: circularity (K); irregularity (L), defined as a combined measure of circularity, shape index, and solidity; and size (M), defined by area, perimeter, and other dimensional parameters. These illustrations emphasize the fact that while static snapshots of different neutrophils at specific timepoints may reveal similar cell shapes, their dynamic shape changes can differ significantly. In all graphs, colored dots represent individual neutrophils (*N* = 177 for CTRL, 89 for DEX, and 127 for LPS) from 4 combined ventricles per group; the black horizontal bars represent the corresponding mean. Black diamonds represent mean values per ventricle. One-way ANOVA and Tukey's post hoc test for multiple comparisons of individual neutrophils across treatments (*P* values included in the graphs). Complementary per-ventricle statistics are provided in Table S2.

Journal of Cell Science • Supplementary information

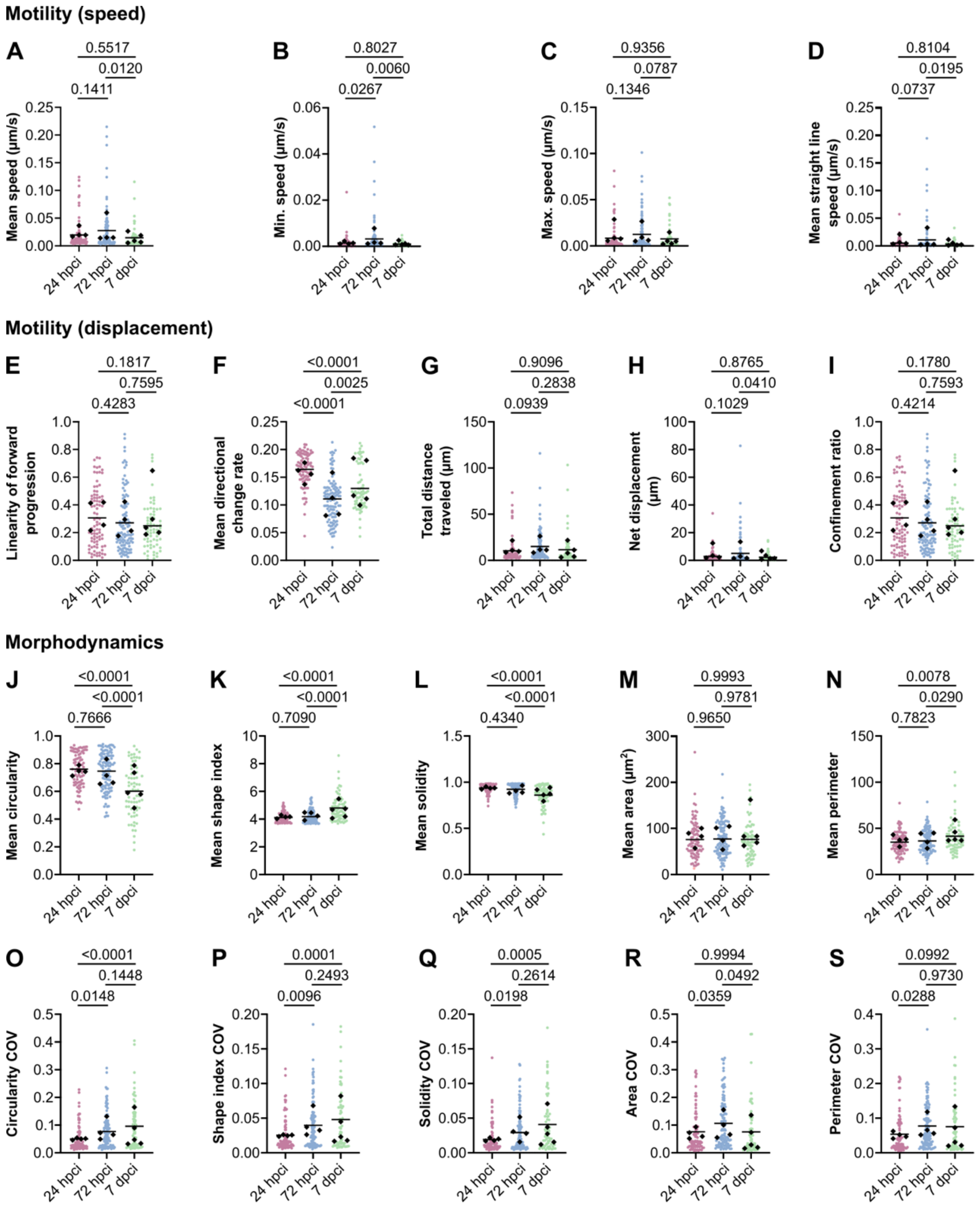

Fig. S5. Detailed analysis of neutrophil motility and morphodynamic parameters during cardiac regeneration. (**A-S**) Beeswarm plots showing motility speed (A-D) and displacement (E-I), and morphodynamic (J-S) parameters in neutrophils from cryoinjured ventricles at 3 different regenerative stages. In all graphs, colored dots represent individual neutrophils (*N* = 86 for 24 hpci, 113 for 72 hpci, 69 for 7 dpci) from 4-5 combined ventricles per group; the black horizontal bars represent the corresponding mean. Black diamonds represent mean values per ventricle. One-way ANOVA and Tukey's post hoc test for multiple comparisons of individual neutrophils across timepoints (*P* values included in the graphs). Complementary per-ventricle statistics are provided in Table S3.

Journal of Cell Science • Supplementary information

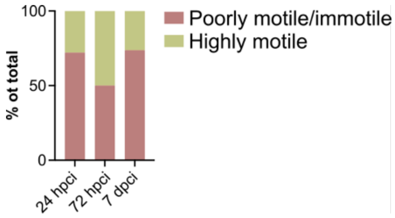

Fig. S6. A larger proportion of highly motile neutrophils is observed at 72 hpci. Quantification of poorly motile/immotile (< 10 μm) and highly motile (> 10 μm) neutrophils across timepoints, based on total distance traveled. Data include all neutrophils (N = 86 for 24 hpci, 113 for 72 hpci, 69 for 7 dpci) from 4-5 combined ventricles per group. Chi-square test (P = 0.0012).

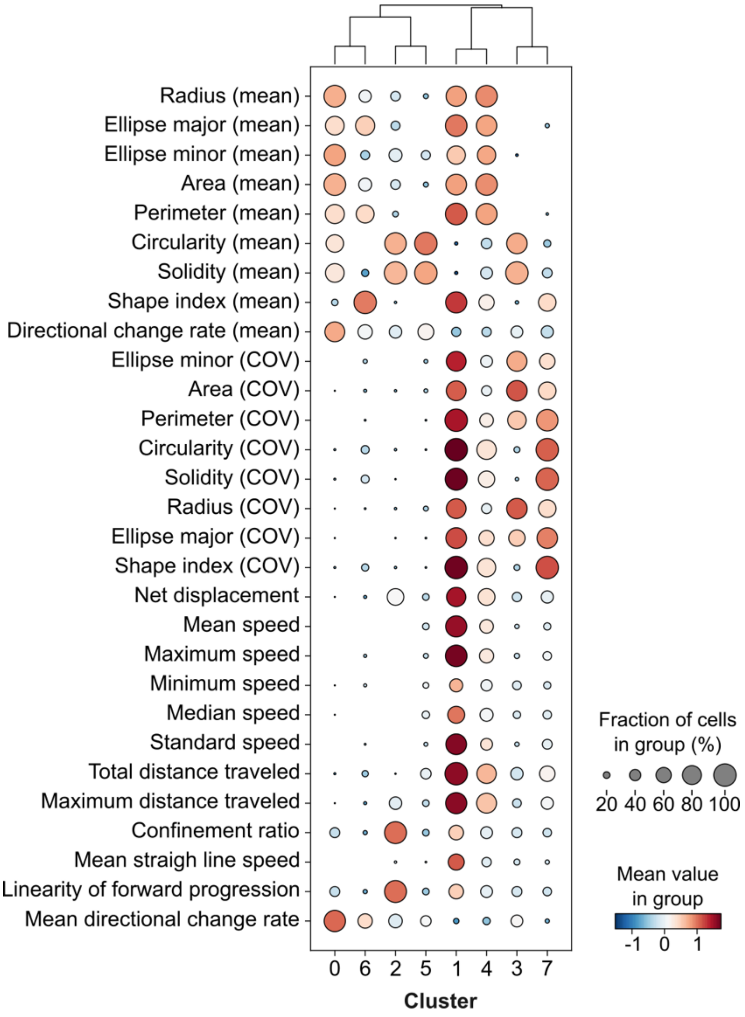

Fig. S7. Different neutrophil behavioral clusters are present in the regenerating zebrafish heart. Dot plot illustrating the enrichment (i.e., mean value and percentage of neutrophils) of the various parameters across clusters. The analysis includes all neutrophils from all ventricles at 24 hpci, 72 hpci, and 7 dpci combined. Clusters are sub grouped according to hierarchical clustering. Data include all neutrophils (*N* = 86 for 24 hpci, 113 for 72 hpci, 69 for 7 dpci) from 4-5 combined ventricles per group.

Journal of Cell Science • Supplementary information

Table S1. Per-ventricle single-parameter (motility) statistics of immunomodulation experiments. Kruskal-Wallis; *P* values are presented.

Parameter	*P* value
Mean speed	0.0031
Min. speed	0.0031
Max. speed	0.0076
Mean straight line speed	0.0048
Linearity of forward progression	0.0194
Mean directional change rate	0.0442
Total distance traveled	0.0031
Net displacement	0.0066
Confinement ratio	0.0194

Table S2. Per-ventricle single-parameter (morphodynamics) statistics of immunomodulation experiments. Kruskal-Wallis; *P* values are presented.

Parameter	*P* value
Mean circularity	0.4360
Mean shape index	0.4516
Mean solidity	0.3967
Mean area	0.1042
Mean perimeter	0.1139
Circularity COV	0.0242
Shape index COV	0.0242
Solidity COV	0.0680
Area COV	0.0403
Perimeter COV	0.0327

Table S3. Per-ventricle single-parameter (motility and morphodynamics) statistics of regeneration experiments. Kruskal-Wallis; *P* values are presented.

Parameter	*P* value
Mean speed	0.2445
Min. speed	0.1764
Max. speed	0.1213
Mean straight line speed	0.2787
Linearity of forward progression	0.7978
Mean directional change rate	0.2136
Total distance traveled	0.3158
Net displacement	0.4652
Confinement ratio	0.7978
Mean circularity	0.2744
Mean shape index	0.2787
Mean solidity	0.2003
Mean area	0.9738
Mean perimeter	0.7916
Circularity COV	0.3047
Shape index COV	0.2488
Solidity COV	0.5885
Area COV	0.4216
Perimeter COV	0.4928

Table S4. Complete set of data of all analyzed neutrophils from freshly collected cryoinjured ventricles and cryoinjured ventricles following 3 h of incubation in control medium.

Available for download at
https://journals.biologists.com/jcs/article-lookup/doi/10.1242/jcs.264418#supplementary-data

Table S5. Complete set of data of all analyzed neutrophils from explanted cryoinjured ventricles upon incubation in control medium (CTRL), DEX or LPS.

Available for download at
https://journals.biologists.com/jcs/article-lookup/doi/10.1242/jcs.264418#supplementary-data

Table S6. Complete set of data of all analyzed neutrophils from cryoinjured ventricles collected at 24 hpci, 72 hpci and 7 dpci.

Available for download at
https://journals.biologists.com/jcs/article-lookup/doi/10.1242/jcs.264418#supplementary-data

Journal of Cell Science • Supplementary information

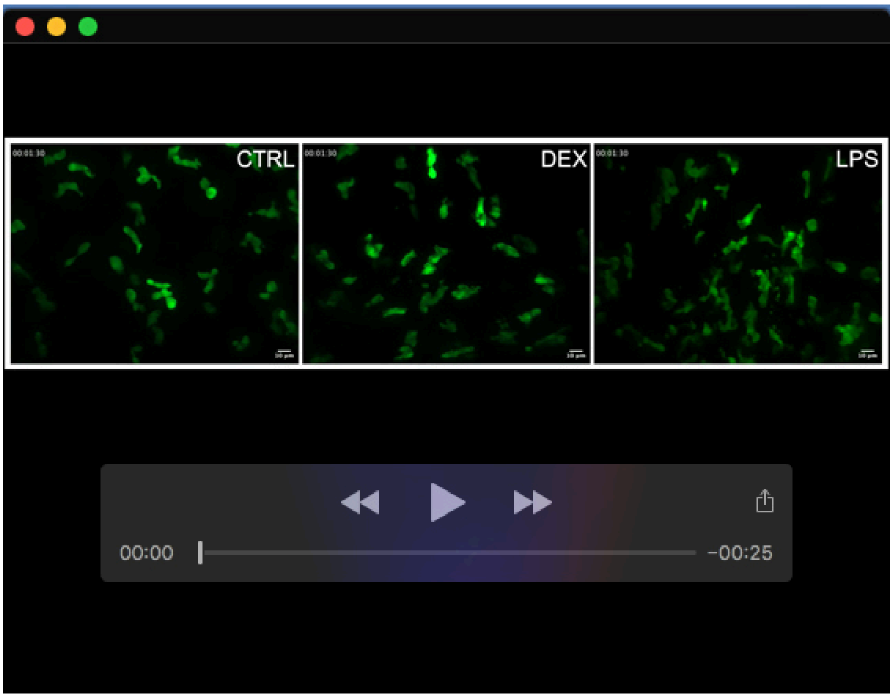

Movie 1. Representative videos showing neutrophils in explanted cryoinjured hearts upon incubation in control medium (CTRL), DEX, or LPS. The movie is a maximum projection of a full Z-stack. Stills of this movie are shown in Fig. 1B. Scale bar is represented. Time is in h:min:s.

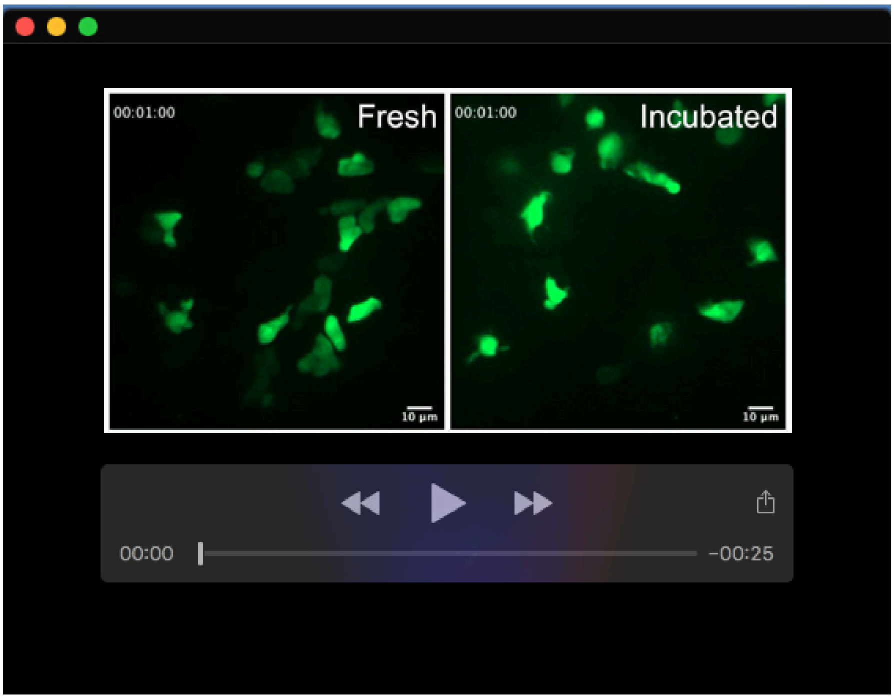

Movie 2. Representative videos showing neutrophils in freshly collected cryoinjured cardiac ventricles and cryoinjured ventricles following 3 hours of incubation in control medium. The movie is a maximum projection of a full Z-stack. Scale bar is represented. Time is in h:min.s.

Journal of Cell Science • Supplementary information

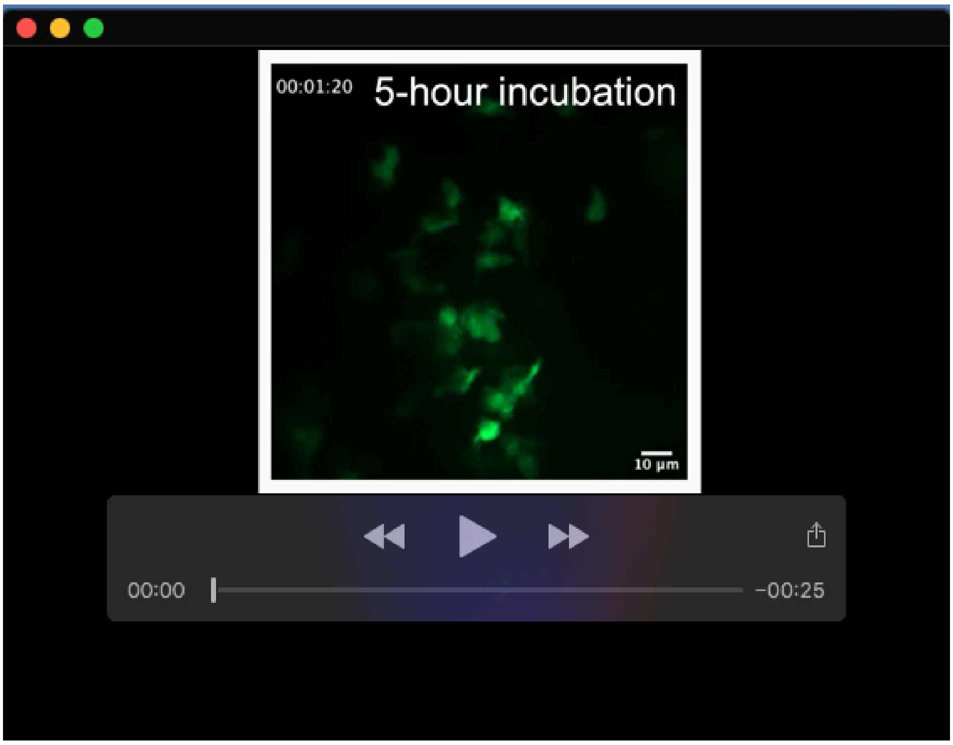

Movie 3. Representative video showing neutrophils in an explanted cryoinjured heart after 5 hours of incubation in control medium. The movie is a maximum projection of a full Z-stack, and is representative from *N* = 4 explants. Scale bar is represented. Time is in h:min.s.

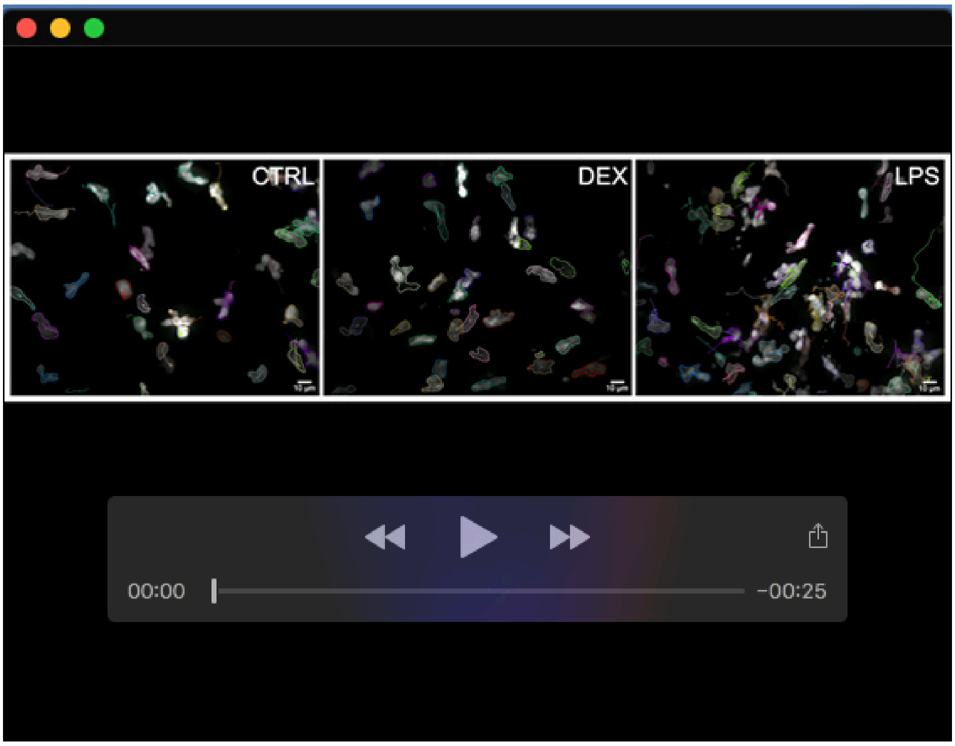

Movie 4. Representative videos showing neutrophils in explanted cryoinjured hearts upon incubation in control medium (CTRL), DEX, or LPS, following segmentation. This movie shows the same videos as in Movie 1, with neutrophil segmentation and tracks represented by the colored lines. Stills of this movie are shown in Fig. 2A. Scale bar is represented.

Journal of Cell Science • Supplementary information

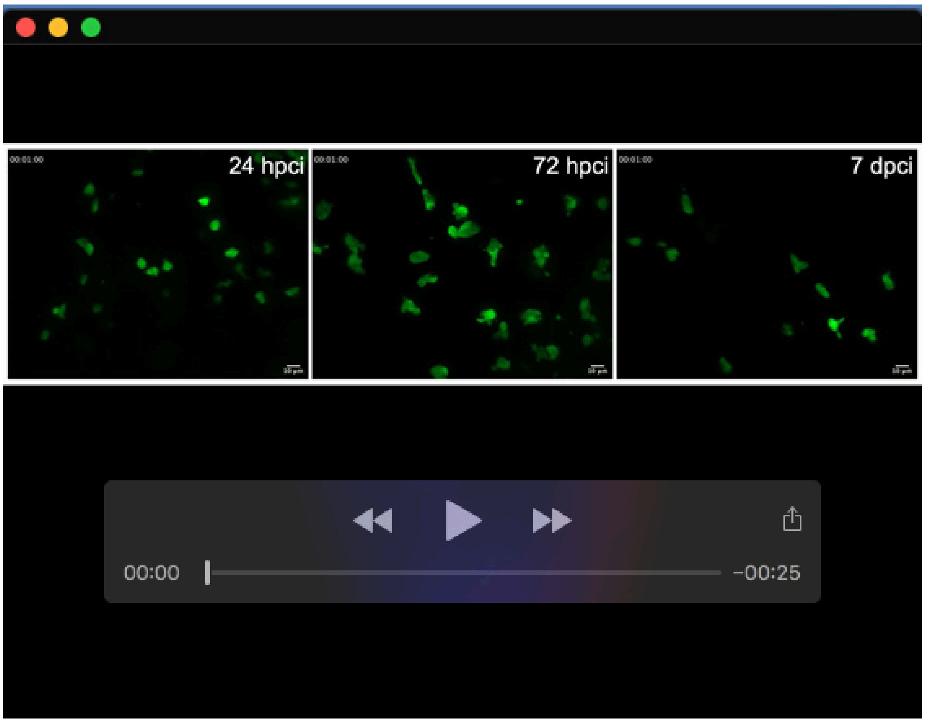

Movie 5. Representative videos showing neutrophils in cryoinjured cardiac ventricles collected at 24 hpci, 72 hpci, and 7 dpci. The movie is a maximum projection of a full Z-stack. Stills of this movie are shown in Fig. 4A. Scale bar is represented. Time is in h:min:s.

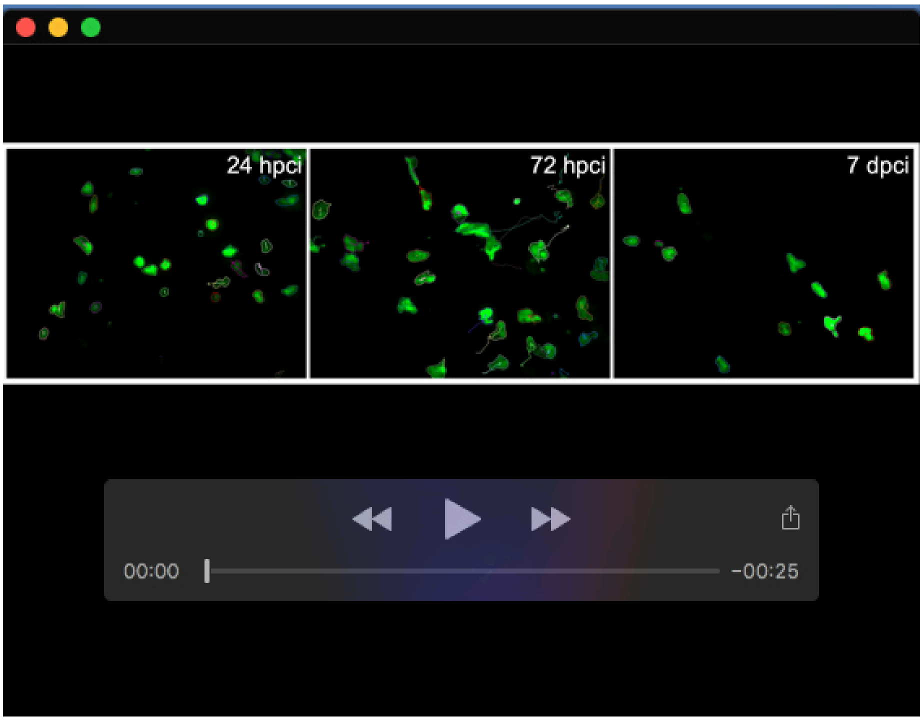

Movie 6. Representative videos showing neutrophils in cryoinjured cardiac ventricles collected at 24 hpci, 72 hpci, and 7 dpci, following segmentation. This movie shows the same videos as in Movie 5, with neutrophil segmentation and tracks represented by the colored lines. Stills of this movie are shown in Fig. 4A. Scale bar is represented.

Journal of Cell Science • Supplementary information
